# Optimization of Very Low-Dose Formulation of Vitamin D3 with Lyophilizate for Dry Powder Inhalation System by Simple Method Based on Time-of-Flight Theory

**DOI:** 10.3390/pharmaceutics13050632

**Published:** 2021-04-29

**Authors:** Kahori Miyamoto, Misato Yanagisawa, Hiroaki Taga, Hiromichi Yamaji, Tomomi Akita, Chikamasa Yamashita

**Affiliations:** Department of Pharmaceutics and Drug Delivery, Faculty of Pharmaceutical Sciences, Tokyo University of Science, 2641 Yamazaki, Noda, Chiba 278-8510, Japan; 3A17706@ed.tus.ac.jp (K.M.); 3B15653@ed.tus.ac.jp (M.Y.); j3a11051@ed.tus.ac.jp (H.T.); 3A14114@ed.tus.ac.jp (H.Y.); akitat@rs.tus.ac.jp (T.A.)

**Keywords:** vitamin D3, dry powder inhalation, pulmonary administration, time-of-flight measurement

## Abstract

It has been previously reported that active vitamin D3 (VD3) is a candidate drug that can repair alveolar damage in chronic obstructive pulmonary disease at a very low dose. We herein report the optimization of a very low-dose formulation of VD3 for dry powder inhalation by a simple method based on time-of-flight (TOF) theory. As the preparation content of VD3 is very low, aerodynamic particle size distribution cannot be measured by pharmacopeial methods that require quantification of the main drug. Thus, a simple method based on TOF theory, which can measure aerodynamic particle size distribution without quantification, was used. The optimized formulation for an inhalation system using a lyophilized cake contained phenylalanine as the excipient (VD3 1 μg/vial + phenylalanine 0.3 mg/vial) and showed high performance with fine particle fraction ≤ 3 μm = 47.2 ± 4.4%. The difference between the results of pharmacopeial methods and simple method was examined using the formulation containing 10 µg/vial of VD3 and was within 5.0%. The preparation is expected to efficiently deliver VD3 to the lungs. Our simple method can optimize dry powder inhalation formulations more easily and rapidly even when the content of the main drug in a preparation is very low.

## 1. Introduction

Chronic obstructive pulmonary disease (COPD) is the third leading cause of death worldwide and is estimated to affect approximately 200 million patients [1,2]. However, no therapeutic drug is currently available to completely cure COPD. We previously showed that active vitamin D3 (VD3) is a candidate drug for alveolar repair that can be used to treat the pathological changes of COPD associated with alveolar destruction [3,4]. Surprisingly, the pulmonary administration of 0.1 μg/kg of VD3 repaired alveoli and improved the respiratory function of COPD model mice.

On the basis of these prior studies, we then aimed to develop VD3 formulations as a treatment for COPD. Dry powder inhalers (DPIs) are commonly used in the treatment of COPD as drugs can be directly delivered deep into the lung with these devices [5,6]. Drugs must be converted into fine particles for delivery using DPIs [7,8,9]. Jet milling or spray drying is widely used in the formulation of drugs for DPIs, but fine particles prepared by these methods have poor flowability [10]. Thus, to improve flowability, fine particles are attached to a lactose carrier or are granulated [8]. For VD3 inhalation formulations, it is difficult to ensure content uniformity with the conventional methods described above because the drug content is very low [11]. We therefore focused on the dry powder inhalation system developed by Yamashita et al., termed lyophilizate for dry powder inhalation (LDPI) [12,13,14]. In the LDPI system, a freeze-dried cake with a porous matrix structure is broken into particles suitable for pulmonary administration by the impact of air introduced in synchronization with the patient’s inspiration (Figure S1). LDPI formulations are prepared by a very simple method of lyophilization, in which a glass vial is filled with the preparation solution, which is then freeze-dried. Thus, the LDPI formulation shows a high degree of content uniformity, even if the drug content is low.

The aerodynamic particle size distribution needs to be measured for optimization of formulations for DPIs since it links to the lung deposition [11,15]. In pharmacopeia, inhalation characteristic tests using impactors (e.g., cascade impactor and multi-stage liquid impinger [MSLI]) which require quantification of the main drug by high-performance liquid chromatography (HPLC) or other modalities are listed as measurement methods of the aerodynamic particle size distribution. However, considering the limits of quantitation [16,17,18], we could not measure the aerodynamic particle size distribution by these methods, as the VD3 content of the formulation we aimed to develop is very low. In fact, at least 100 vials of low-dose LDPI formulations of VD3 would be needed to perform the inhalation characteristic test by pharmacopeial methods, and this is not realistic. As just described, to optimize the very low-dose formulation of VD3, a method must be devised to measure the aerodynamic particle size distribution. Thus, we decided to measure this distribution by a method that does not require quantification of the main drug. Time-of-flight (TOF) measurement is one method that can measure the aerodynamic particle size distribution without requiring quantification of the main drug [19,20]. We have already developed a measurement method based on TOF theory [21]. Our method has following two features: (1) the dispersion condition is same as for MSLI measurement, that is formulations are aerosolized under the condition which reflects human inhalation. (2) Measurement of the aerodynamic size distribution of particles generated under the condition described just above can be performed in short time by TOF. In this simple method based on TOF theory, a result from which the results of MSLI measurement can be estimated is obtained; that is, our method can be used as a surrogate method for MSLI.

From this background, we aimed to develop a very low-dose dry powder formulation of VD3, a candidate drug for alveolar repair in COPD, according to the following strategy. We prepare VD3 formulations with the LDPI system to ensure content uniformity. LDPI formulations of VD3 are optimized by a simple method based on TOF theory to measure the aerodynamic particle size distribution without quantification of VD3.

## 2. Materials and Methods

### 2.1. Materials

Materials used in this study were purchased from the following commercial vendors: VD3 from Cayman Chemical (Ann Arbor, MI, USA). Excipients (special grade)—L-alanine (Ala), L-methionine (Met), L-phenylalanine (Phe), L-leucine (Leu), L-valine (Val) and L-isoleucine (Ile)—and reagents—ethanol (99.5%) (reagent grade), acetonitrile (HPLC grade) and polyoxyethylene (20) sorbitan monolaurate (biochemistry grade)—from Fujifilm Wako Pure Chemical Industries (Osaka, Japan). 2 mL VIST glass vials obtained from Daiwa Special Glass (Osaka, Japan) and rubber stoppers (F5-43) obtained from Sumitomo Rubber Industries (Hyogo, Japan) were used as the packing materials.

### 2.2. Preparation of Freeze-Dried Cake for Dry Powder Inhalation of VD3

For the formulation of VD3, the human equivalent dose was calculated in the manner determined by FDA guidance [22], i.e., as 0.1 × 0.08 = 0.008 μg/kg from the dosage in mice of 0.1 µg/kg. The clinical dose when the human body weight is set to 60 kg is 0.5 µg/day. Assuming that the efficiency of delivery to the lung is 50%, we set the VD3 content of the formulation developed in this study to 1 µg.

Stock solutions of amino acids were prepared by dissolving them in purified water (2–16 mg/mL). VD3 was dissolved in ethanol (0.2 or 2 mg/mL) and then suspended in diluted stock solutions of amino acids to obtain the target concentration (VD3 2 µg/mL with amino acid 0.2–1.6 mg/mL or VD3 20 µg/mL with Phe 0.6 mg/mL). Each vial was filled with 500 µL of the suspension and frozen using liquid nitrogen. Then, lyophilization was carried out in a benchtop freeze dryer (FreeZone Triad 7400030, LABCONCO, Kansas City, MO, USA). Shelf was precooled at −50 °C, and shelf temperature was increased to −30 °C at pressure of 1.0 Pa for primary drying. After primary drying for 11 h, secondary drying was performed at shelf temperature of 35 °C (shelf was ramped at 0.17 °C/min) for 5 h. Finally, shelf temperature was decreased to 25 °C at ramp rate of −0.34 °C/min and held at 25 °C for 1 h.

### 2.3. Visual Evaluation of the Cake Appearance

In lyophilized formulations, the lyophilized cake is often required to have a good appearance without cracks or chips [23]. The LDPI system also requires a freeze-dried cake with good appearance to ensure stable performance. Thus, the cake appearance was evaluated by configuration score.

The freeze-dried cake was classified into three types according to appearance with a configuration score. A configuration score of 0 indicates that no cake was formed, a score of 1 indicates that the appearance of the cake was not suitable because cracks or chips were observed and a score of 2 indicates that a cake without cracks or chips was formed and that its appearance was suitable for the LDPI formulation.

### 2.4. Measurement of Aerodynamic Particle Size Distribution

When measuring aerodynamic particle size distribution, it would be ideal to perform an inhalation characteristic test by pharmacopeial methods. However, as the preparation content of VD3 is very low, it is difficult to test the inhalation characteristics, which is necessary to quantify the main drug by HPLC or other modalities [16,17]. TOF measurement is one method that can directly measure the aerodynamic particle size distribution without requiring quantification of the main drug, and thus, TOF measurement was mainly performed for optimization.

To evaluate the aerosolization performance of formulations, the fine particle fraction (FPF) ≤ 3 μm calculated from the aerodynamic particle size distribution was used. The aim of the VD3 formulation is treatment of COPD by alveolar repair. As the target of VD3 is the alveolar region, VD3 should be delivered to alveoli deep within the lungs. In the DPI formulations, aerosols must have aerodynamic diameters of ≤3 µm to ensure drug delivery to the alveoli [24].

#### 2.4.1. TOF Measurement

TOF measurement was performed with an Aerodynamic Particle Sizer (APS; Model 3321, TSI, Shoreview, MN, USA). We have developed a measurement method using the APS in which the APS results can be used to estimate the results of MSLI [21], and APS measurement was performed by this method (Figure S2). Briefly, a custom-made glass throat and a linear vacuum pump (VP0940, Nitto Kohki, Tokyo, Japan) were added to APS system to disperse formulations under the condition of flow rate at 30 L/min with two-way needle device given a pressure drop of 4 kPa, the same as for MSLI measurement. Particles generated under the above condition were diluted with aerosol diluter of the APS (Model 3302A, TSI) and introduced into a model 3321 APS spectrometer (TSI) at flow rate of 5 L/min, the standard condition for APS measurement. TOF measurements were done every 1 s for 8 s and the particle size distribution was calculated. The percentage of particles of ≤3 μm in cumulative mass-weighted distribution was defined as APS FPF ≤ 3 μm. Each sample was measured in triplicate.

#### 2.4.2. In Vitro Inhalation Characteristic Test by MSLI

Inhalation performance was characterized using MSLI. Test condition was maintained flow rate of 30 ± 0.3 L/min and pressure drop of 4 kPa with two-way needle device (Otsuka, Tokyo, Japan) using a vacuum pump (HCP5), critical flow controller (TPK2000) and flow meter (DFM2000; all from Copley Scientific Limited, Nottingham, UK). Effective cut-off diameters for stages 2–4 were 9.6, 4.4 and 2.4 µm, respectively, under the above test condition. A filter having a retention diameter of 0.65 µm (hydrophilic poly (vinylidene fluoride) membrane; Merck Millipore, Burlington, MA, USA) was placed at stage 5. In this case, 10 vials of formulations containing 10 µg/vial of VD3 were used per experiment and each vial of formulations was aerosolized for 8 s under the above test condition. Each deposition experiment was repeated in triplicate.

VD3 was extracted into the diluent (20% acetonitrile solution containing 0.1% polyoxyethylene (20) sorbitan monolaurate) from each of stages 1–5 of the MSLI, the vial, device and induction port. Extracted VD3 was quantified by HPLC (Prominence series, Shimadzu, Kyoto, Japan). Measurements were performed at flow rate of 1.0 mL/min and a column temperature of 40 °C. A YMC-Pack ODS-AQ column (150 × 4.6 mm; YMC, Kyoto, Japan) was used and 100 µL of the sample solution was applied. Water and acetonitrile were used as the mobile phase and their concentration gradient was controlled as follows: 0 min, 20% acetonitrile; 30 min, 100% acetonitrile. VD3 was detected by a UV absorption photometer (wavelength 265 nm).

From the VD3 mass of each of stages 1–5 of the MSLI, the vial, device and induction port, the emission and MSLI FPF ≤ 3 μm were calculated. The emission was the ratio of the sum of the VD3 mass of each stages of 1–5 of the MSLI and induction port (the emitted dose) to VD3 content of the formulation. MSLI FPF ≤ 3 μm was the ratio of the cumulative mass proportion of VD3 of ≤3 μm to the emission.

### 2.5. Scanning Electron Microscopy

The microstructure of the lyophilized cake was observed by scanning electron microscopy (SEM, JSM-6060LA, JEOL, Tokyo, Japan) at 15 kV. The samples were sputtered with platinum under vacuum using sputtering equipment (JFC-1600, JEOL).

### 2.6. X-ray Diffractometry (XRD)

The lyophilized cakes were investigated with an RINT-2000 X-ray diffractometer (Rigaku, Tokyo, Japan) equipped with a copper anode (40 kV, 40 mA). The samples were measured from 5 to 40° at a step rate of 2*θ* = 0.02° with 0.12 s measurement time per step.

## 3. Results

### 3.1. Selection of Excipients for LDPI Formulations

Excipients play an important role in the generation of particles suitable for inhalation in the LDPI system. In comparison to saccharides, which are widely used as excipients for freeze-dried preparations, amino acids (especially hydrophobic amino acids) showed high aerosolization performance as excipients for LDPI formulations [25,26,27]. Therefore, we performed the screening study focusing on hydrophobic amino acids rather than saccharides.

In lyophilized formulations, the lyophilized cake is often required to have a good appearance without cracks or chipping [23]. In the LDPI system, a lyophilized cake is aerosolized just upon inhalation by air impact. When the cake appearance is poor, aerosolization performance on inhalation may fluctuate due to static generation among fragmented pieces of the cake [28]. Thus, good cake appearance is necessary to guarantee stable aerosolization performance of LDPI formulations.

For the above reasons, measurement of APS FPF ≤ 3 μm and visual evaluation of the cake appearance were performed using formulations containing 1 µg/vial of VD3 and 0.1, 0.2 or 0.4 mg/vial of hydrophobic amino acid, for which the hydropathy index is a positive value (Ile: 4.5, Val: 4.2, Leu: 3.8, Phe: 2.8, Met: 1.9, Ala: 1.8; [29]), to select the optimum amino acid for the excipient.

**Figure 1:**
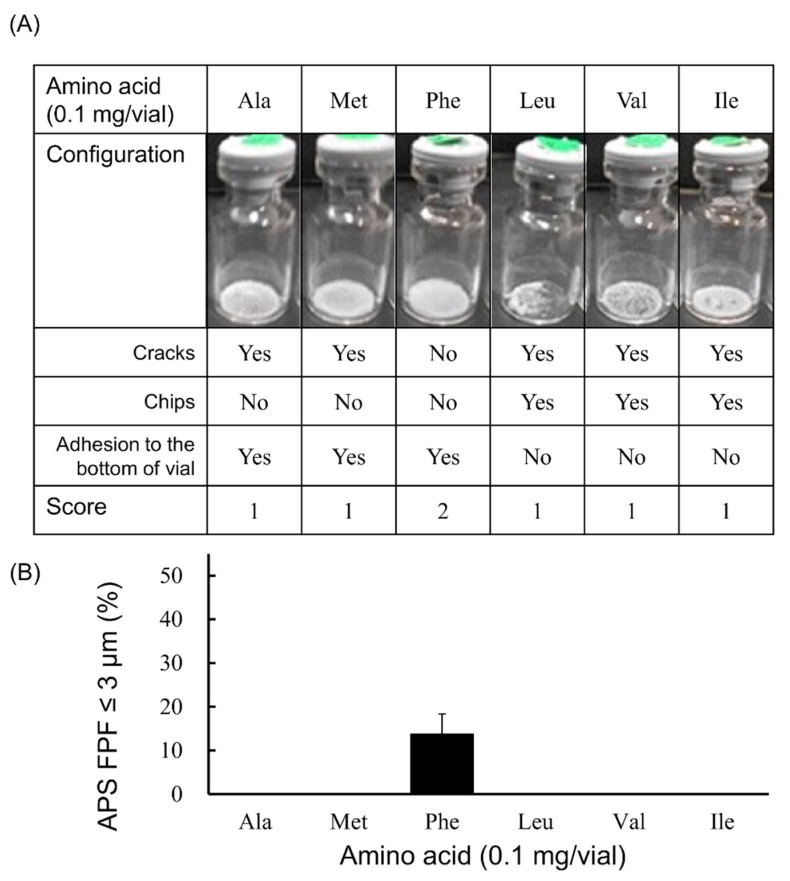
Evaluation of configuration and aerosolization performance of VD3 formulations added 0.1 mg/vial of hydrophobic amino acids using with the LDPI system. Representative photographs of formulations, detailed description of cake appearance and configuration score are shown in (**A**). Values of APS FPF ≤ 3 μm (mean ± SD, *n* = 3) are shown in (**B**). VD3 content of formulations was 1 µg/vial. Any of the following hydrophobic amino acids: alanine (Ala), methionine (Met), phenylalanine (Phe), leucine (Leu), valine (Val) or isoleucine (Ile) was added. APS, aerodynamic particle sizer; FPF, fine particle fraction.

In the case that 0.1 mg/vial of amino acid was added (Figure 1), the generation of fine particles of ≤3 µm was observed only in the Phe-added formulation, and the APS FPF ≤ 3 μm was 13.8 ± 4.6%. In addition, only the cake from the Phe-added formulation had a configuration score of 2. The cakes of the other formulations in which Ala, Met, Leu, Val or Ile was added had a configuration score of 1.

**Figure 2:**
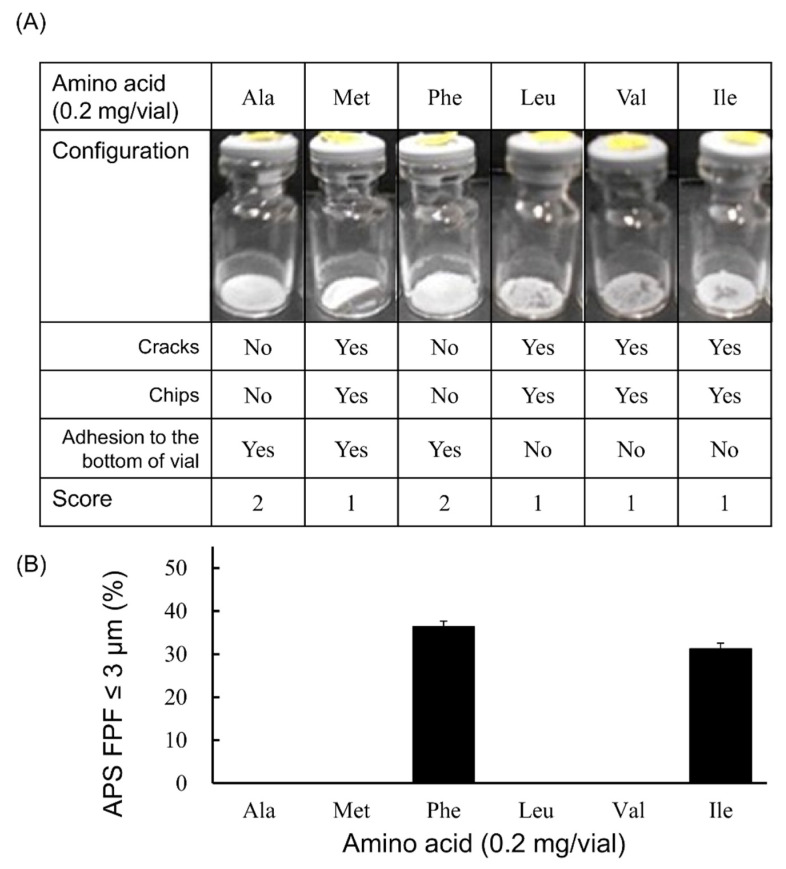
Evaluation of configuration and aerosolization performance of VD3 formulations added 0.2 mg/vial of hydrophobic amino acids using with the LDPI system. Representative photographs of formulations, detailed description of cake appearance and configuration score are shown in (**A**). Values of APS FPF ≤ 3 μm (mean ± SD, *n* = 3) are shown in (**B**). VD3 content of formulations was 1 µg/vial. Any of the following hydrophobic amino acids: alanine (Ala), methionine (Met), phenylalanine (Phe), leucine (Leu), valine (Val) or isoleucine (Ile) was added. APS, aerodynamic particle sizer; FPF, fine particle fraction.

Among the amino acid-added formulations of 0.2 mg/vial (Figure 2), the formulation in which Phe or Ile was added showed the generation of fine particles of ≤3 µm. The APS FPF ≤ 3 μm was 36.4 ± 1.3% for the Phe-added formulation and 31.3 ± 1.3% for the Ile-added formulation. Regarding the appearance of the freeze-dried cake, the configuration score was 2 in the Phe-added formulation, whereas that for the Ile-added formulation was 1. Apart from Phe, the cake of the formulation in which Ala was added also had a configuration score of 2, but the generation of fine particles of ≤3 µm was not observed.

**Figure 3:**
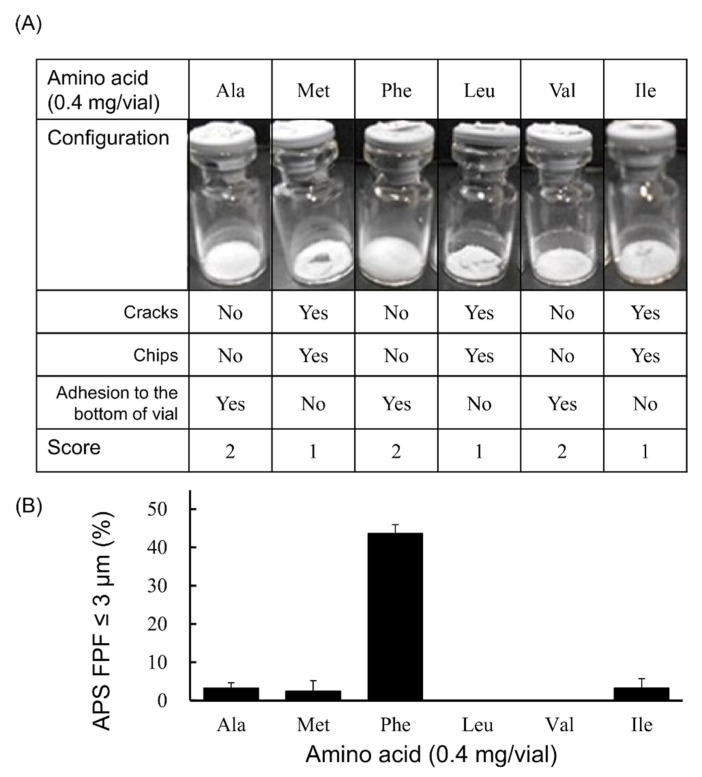
Evaluation of configuration and aerosolization performance of VD3 formulations added 0.4 mg/vial of hydrophobic amino acids used with the LDPI system. Representative photographs of formulations, detailed description of cake appearance and configuration score are shown in (**A**). Values of APS FPF ≤ 3 μm (mean ± SD, *n* = 3) are shown in (**B**). VD3 content of formulations was 1 µg/vial. Any of the following hydrophobic amino acids: alanine (Ala), methionine (Met), phenylalanine (Phe), leucine (Leu), valine (Val) or isoleucine (Ile) was added. APS, aerodynamic particle sizer; FPF, fine particle fraction.

In the case that the added amount was 0.4 mg/vial (Figure 3), fine particles of ≤3 µm were generated by adding any of these 4 types of amino acids (Ala, Met, Phe or Ile). Among these 4 amino acids, APS FPF ≤ 3 μm of the Phe-added formulation was particularly high, at 43.7 ± 2.3%. The configuration score was 2 in the cake of the formulation to which Ala or Phe was added. However, the formulation to which Met or Ile was added had a configuration score of 1.

**Figure 4:**
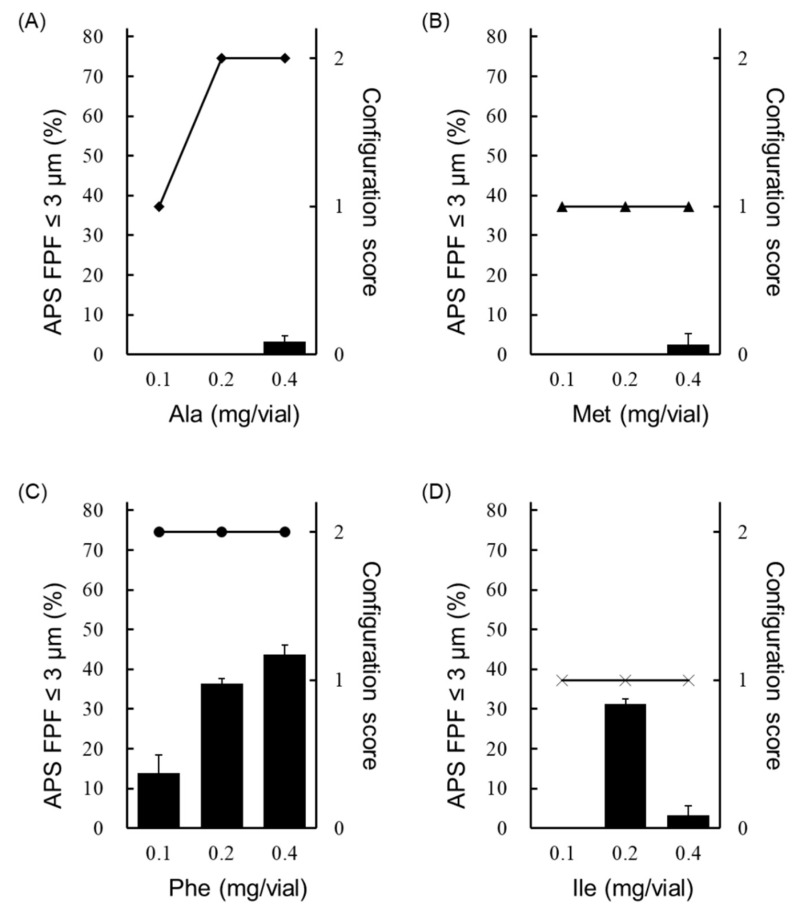
Effect of the added amount of hydrophobic amino acids on the configuration score and FPF in the LDPI system. Configuration scores are shown as symbols, and values of APS FPF% ≤ 3 μm (mean ± SD, *n* = 3) are shown as black bars. Graphs show the configuration score and values of APS FPF ≤3 μm of the (**A**) Ala-added formulation, (**B**) Met-added formulation, (**C**) Phe-added formulation and (**D**) Ile-added formulation. Formulations were prepared to contain 1 µg/vial of VD3 with 0.1, 0.2 or 0.4 mg/vial of amino acid. APS, aerodynamic particle sizer; FPF, fine particle fraction; Ala, alanine; Met, methionine; Phe, phenylalanine; Ile, isoleucine.

Next, the effect of the added amount of amino acids (Ala, Met, Phe and Ile) that showed the generation of fine particles of ≤3 µm on APS FPF ≤ 3 μm and the configuration score was examined (Figure 4). Only the Phe-added formulation showed the generation of fine particles of ≤3 µm in every case with the added amount of 0.1, 0.2 or 0.4 mg/vial (Figure 4C). Moreover, the Phe-added formulation showed higher APS FPF ≤ 3 μm than formulations to which Ala, Met or Ile was added, and the APS FPF ≤ 3 μm was highest when 0.4 mg/vial of Phe was added. The Ile-added formulation showed high APS FPF ≤ 3 μm at the added amount of 0.2 mg/vial, but it showed a substantial decrease of APS FPF ≤ 3 μm as the added amount was increased (Figure 4D). Formulations to which Ala or Met was added showed the generation of fine particle of ≤3 µm only when the added amount was 0.4 mg/vial, and the APS FPF ≤ 3 μm was low (approximately 3%; Figure 4A,B). Regarding cake appearance, the configuration score of the Phe-added formulation was 2 regardless of the amount added (Figure 4C). The configuration score of the formulation to which Met or Ile was added was also unchanged when the added amount increased, but the score was 1 (Figure 4B,D). In contrast, the formulation with Ala added showed an increase in the configuration score as the added amount was increased (Figure 4A).

From the above results, Phe was selected as the optimum excipient, and its optimum amount was examined.

### 3.2. Optimization of Phe Amount in LDPI Formulation

**Figure 5:**
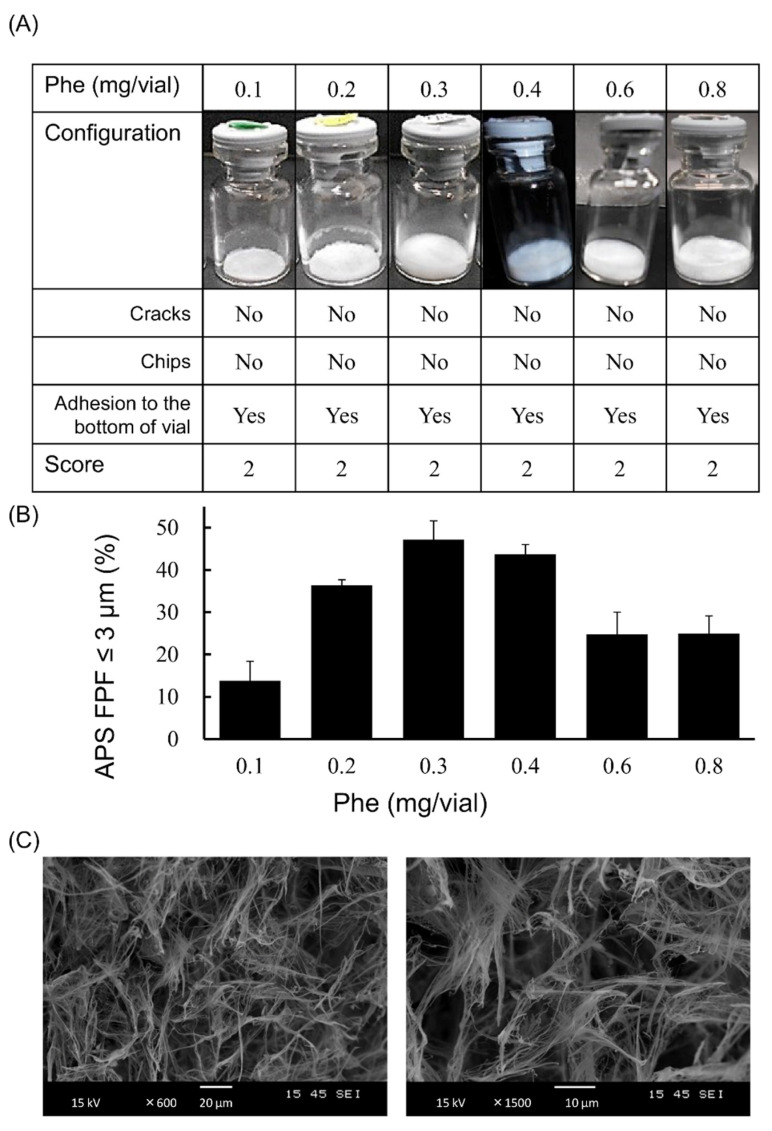
Optimization of the added amount of Phe in very low-dose formulations of VD3 using with the LDPI system. Representative photographs of formulations, detailed description of cake appearance and configuration score are shown in (**A**). Values of APS FPF ≤ 3 μm (mean ± SD, *n* = 3) are shown in (**B**). VD3 content of formulations was 1 µg/vial. The added amount of Phe was 0.1, 0.2, 0.3, 0.4, 0.5, 0.6 or 0.8 mg/vial. The microstructure of the formulation with 0.3 mg/vial of Phe added as observed by scanning electron microscope is shown in (**C**). APS, aerodynamic particle sizer; FPF, fine particle fraction; Phe, phenylalanine.

Phe, which showed both high aerosolization performance and good cake appearance, was selected as the optimum excipient. To optimize the added amount of Phe, measurement of APS FPF ≤ 3 μm and visual evaluation of the cake appearance were performed using formulations containing 1 µg/vial of VD3 with 0.1, 0.2, 0.3, 0.4, 0.6 or 0.8 mg/vial of Phe. A lyophilized cake with configuration score 2 was formed regardless of the amount of Phe added (Figure 5A). The APS FPF ≤ 3 μm of the formulation to which 0.3 mg/vial of Phe was added was the highest (47.2 ± 4.4%, Figure 5B). Additionally, the microstructure of the lyophilized cake was observed by SEM. The cake showed a porous matrix with fiber-like network structure (Figure 5C).

### 3.3. In Vitro Inhalation Characteristics of VD3 Formulations with LDPI System by MSLI

**Figure 6:**
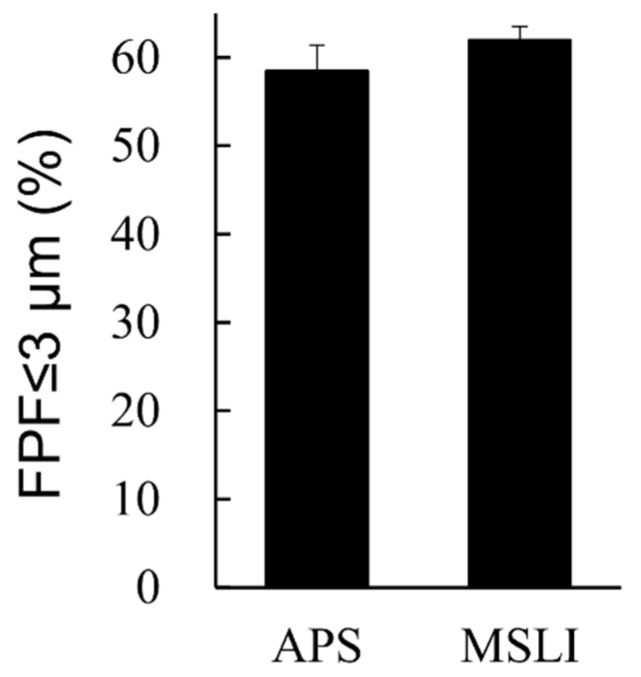
Comparison of results by MSLI versus APS using formulations containing 10 times the clinical estimated dosage of VD3. Values of FPF ≤ 3 μm measured by APS or MSLI are shown (mean ± SD, *n* = 3). Formulations were prepared to contain 10 µg/vial of VD3 with 0.3 mg/vial of phenylalanine. MSLI, multi-stage liquid impinger; APS, aerodynamic particle sizer; FPF, fine particle fraction.

The formulation we aimed to developed has a very low VD3 content of 1 µg/vial. Considering the limits of quantitation, we were unable to measure the inhalation characteristics of the formulation containing VD3 1 µg/vial by MSLI. Therefore, a formulation containing 10 times the amount of VD3 (VD3 10 µg/vial + Phe 0.3 mg/vial) was prepared, and the FPF was measured by APS and MSLI. As a result of comparing FPF ≤ 3 μm between APS and MSLI, no significant difference was observed (Figure 6).

### 3.4. Crystallinity of Excipients in LDPI Formulations by XRD

**Figure 7:**
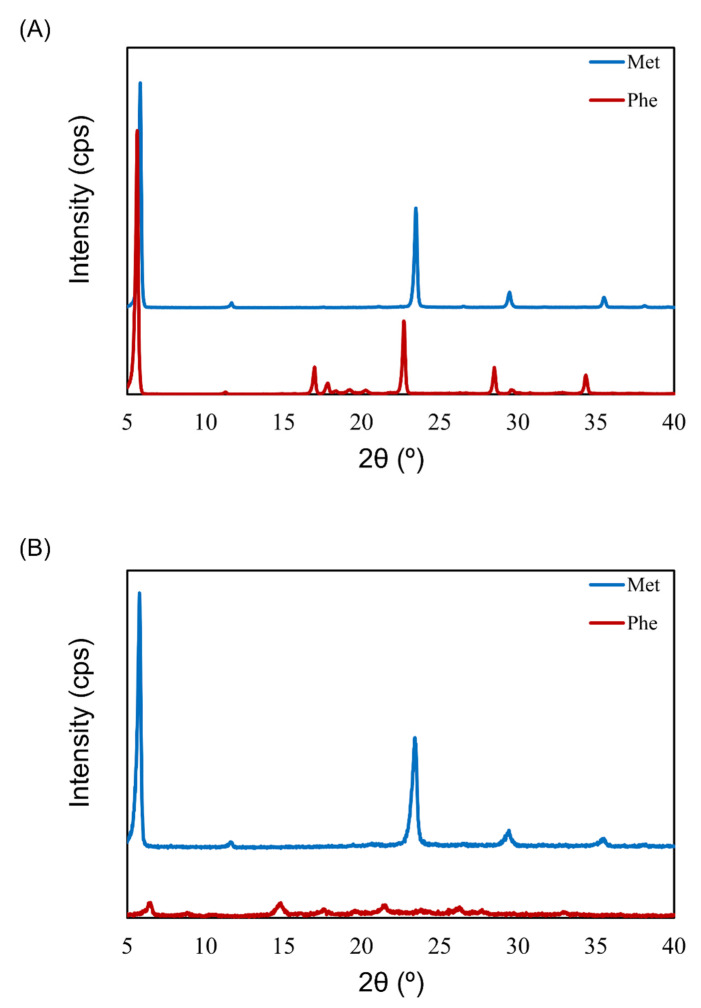
X-ray diffractometry (XRD) patterns before and after freeze-drying of excipients for LDPI formulations. XRD pattern before (**A**) and after (**B**) freeze-drying of Phe or Met. The lyophilizates were prepared to contain 0.5 mg/vial of Phe or Met. Phe, phenylalanine; Met, methionine.

To understand the impact of the physical state of lyophilized cake on aerosolization performance, the crystallinity of different placebo formulations was inspected with XRD. The placebo formulations were single-excipient formulations containing 0.5 mg/vial of Phe or Met. The results of both Phe and Met before freeze-drying showed a crystalline pattern (sharp peaks with strong intensity, Figure 7A). There was no difference in the XRD pattern of Met before and after freeze-drying. However, the XRD pattern of Phe after freeze-drying was different than that before freeze-drying. The lyophilized cake of Phe showed a few broadened peaks with very weak intensity (Figure 7B). The placebo formulations showed the same tendency as the VD3 formulation. Phe showed both good cake appearance (configuration score 2) and high APS FPF ≤ 3 μm (approximately 40%) in contrast to Met (configuration score 1, APS FPF ≤ 3 μm approximately 5%).

## 4. Discussion

In this study, TOF measurement by APS was mainly performed for optimization because the VD3 content of the formulations was very low (1 µg/vial), and the inhalation characteristic test requiring quantification of the main drug could not be performed. While we previously reported that the result of MSLI can be estimated from the result of our method using APS [21], we confirmed that same applies to VD3 using the formulation containing 10 µg/vial of VD3, i.e., 10 times the clinical estimated dosage of 1 µg/vial. The difference between MSLI FPF ≤ 3 μm and APS FPF ≤ 3 μm was within 5.0% (Figure 6). Thus, MSLI FPF ≤ 3 μm of the optimized formulation containing VD3 1 μg/vial and Phe 0.3 mg/vial is expected to be approximately 50% from APS FPF ≤ 3 μm.

In the inhalation characteristic test by MSLI, 10 vials of formulations containing 10 µg/vial of VD3 were used per experiment. If we try to measure the aerodynamic particle size distribution of the formulation containing 1 µg/vial of VD3 by MSLI, at least 100 vials would be needed per experiment, which is not realistic. Additionally, this does not meet various pharmacopeia’s requirement (e.g., European pharmacopeia and Japanese pharmacopeia). The number of discharges is typically required to be ≤10 in such pharmacopeias. However, APS measurement can be performed using one vial per experiment. Furthermore, APS can measure the aerodynamic particle size distribution of many samples in a short time whereas MSLI measurement requires much time even for only a few samples. Our simple method using APS (Figure S2) was shown to be useful as a surrogate method for MSLI to optimize a formulation more easily and rapidly even when the content of the main drug in preparation is so low as to be difficult to quantify.

FPF ≤ 3 μm is an index of the efficiency at which a formulation can reach the alveoli. The FPF ≤ 3 μm of formulations containing VD3 1 μg/vial and Phe 0.3 mg/vial was nearly 50% (Figure 5B) and the FPF ≤ 5 μm, an index of delivery efficiency to the lung, was approximately 80% (data is not shown), indicating that the goal of developing a formulation that facilitates the delivery of 50% of VD3 1 μg by DPI was achieved. The emitted dose of formulations containing 10 µg/vial of VD3 was 8.7 µg/vial in the MSLI measurement. Assuming that nearly 90% of VD3 is emitted (emitted dose 0.9 µg/vial), optimized formulations is expected to deriver approximately 45% of VD3 to alveoli deep within the lungs and 70% to the lungs from FPF. In addition, the FPF ≤ 5 μm of conventional DPIs is 40% or less [30]. We succeeded in developing a formulation that showed higher performance than conventional DPIs.

The formulation of VD3 was optimized by the addition of Phe, which showed both good cake appearance and high aerosolization performance. An aromatic side chain of Phe is thought to be one of the reasons for the good cake appearance. The side chain with aromatic ring was reported to show higher glass transition temperature due to the stacking interaction [31]. High glass transition temperature is one of the important factors that contributes to good cake appearance [23]. Therefore, Phe may show good cake appearance due to its aromatic side chain. Regarding the microstructure of the cakes, the cake formed by Phe showed a porous matrix, but the structure was different from porous lyophilizates formed by saccharides. The cake formed by Phe has a fiber-like network structure (Figure 5C and Figure S3) and is therefore considered to show high aerosolization ability [12,25,26,27,32].

To inspect the aerosolization mechanism of the formulation containing Phe by air impact, its physical properties were investigated. It is clear that aerosolization performance depends on excipients (Figure 1, Figure 2 and Figure 3). Thus, placebo formulations were used for investigation. The excipient Met showed low aerosolization performance (FPF ≤ 3 μm of <10%) and maintained crystallinity after freeze-drying. However, the crystallinity of the excipient Phe, which showed high aerosolization performance (FPF ≤ 3 μm of approximately 40%), dropped notably after freeze-drying (Figure 7A,B). In the previous study by Claus et al. [26], FPF and crystallinity of LDPI formulations containing 6 mg/vial of lysozyme with 1–4 mg/vial of Phe were evaluated. Formulations used in that study contained higher masses of the main drug and Phe than did the formulations used in the present study. While the formulations cannot be compared simply as the formulations used in the present study differed drastically from those in the previous study in terms of content, Phe showed high aerosolization performance in both low-dose and high-dose formulations. Partial crystallization of Phe was observed in the study by Claus et al. The crystallinity of Phe increased as the added amount increased, but a significant decrease in FPF was not detected. Thus, it is thought that not crystallinity but the surface roughness of particles due to partial crystallization of Phe decreased adhesiveness, which led to FPF improvement. From the above, it cannot be concluded that low crystallinity contributes to high aerosolization performance.

Next, considering the cause of the high aerosolization performance of Phe from the viewpoint of the surface activity of amino acids, because Phe has surface activity [33], it is coordinated at the ice/water and air/water interfaces during freezing, thus forming a hydrophobic coating film that is presumed to surround the periphery of the VD3. A similar phenomenon was also reported by Claus et al. [25]. This film may have caused the high aerosolization performance. However, in the case of Met, which has almost no surface activity [34], the aerosolization of Met is lower than that of Phe because a hydrophobic coating film such as that formed with Phe is not formed on the interface during freezing.

## 5. Conclusions

VD3 is a candidate drug that can repair alveolar damage at a very low dose. We optimized a very low-dose formulation of VD3 for the LDPI system by a method using an APS that can measure the aerodynamic particle size distribution without requiring quantification of the main drug. The VD3 formulation was optimized by the addition of Phe. The FPF ≤ 3 μm of the optimum formulation (VD3 1 μg/vial + Phe 0.3 mg/vial) was 47.2 ± 4.4%. The result was high enough to achieve the goal of 50% delivery efficiency to the lung.

## Data Availability

The data presented in this study are available on request from the corresponding author.

## Supplementary Materials

The following are available online at https://www.mdpi.com/article/10.3390/pharmaceutics13050632/s1, Figure S1: Lyophilizate for dry powder inhalation (LDPI) system, Figure S2: Measuring methods for the aerodynamic particle size distribution of LDPI formulations: multi-stage liquid impinger (MSLI) and our simple method based on time-of-flight (TOF) theory, Figure S3: Microstructure of the placebo formulation containing 0.5 mg/vial of phenylalanine or methionine observed by scanning electron microscope (SEM).

## References

[1] Vos T., Abajobir A.A., Abate K.H., Abbafati C., Abbas K.M., Abd-Allah F., Abdulkader R.S., Abdulle A.M., Abebo T.A., Abera S.F. (2017). Global, regional, and national incidence, prevalence, and years lived with disability for 328 diseases and injuries for 195 countries, 1990–2016: A systematic analysis for the Global Burden of Disease Study 2016. Lancet.

[2] Ellingsen J., Johansson G., Larsson K., Lisspers K., Malinovschi A., Ställberg B., Thuresson M., Janson C. (2020). Impact of Comorbidities and Commonly Used Drugs on Mortality in COPD—Real-World Data from a Primary Care Setting. Int. J. Chron. Obstruct. Pulmon. Dis..

[3] Akita T., Hirokawa M., Yamashita C. (2020). The effects of 1α,25-dihydroxyvitamin D3 on alveolar repair and bone mass in adiponectin-deficient mice. J. Steroid Biochem. Mol. Biol..

[4] Horiguchi M., Hirokawa M., Abe K., Kumagai H., Yamashita C. (2016). Pulmonary administration of 1,25-dihydroxyvitamin D3 to the lungs induces alveolar regeneration in a mouse model of chronic obstructive pulmonary disease. J. Control. Release.

[5] Lavorini F., Corrigan C.J., Barnes P.J., Dekhuijzen P.R.N., Levy M.L., Pedersen S., Roche N., Vincken W., Crompton G.K. (2011). Retail sales of inhalation devices in European countries: So much for a global policy. Respir. Med..

[6] Sorino C., Negri S., Spanevello A., Visca D., Scichilone N. (2020). Inhalation therapy devices for the treatment of obstructive lung diseases: The history of inhalers towards the ideal inhaler. Eur. J. Intern. Med..

[7] De Boer A.H., Gjaltema D., Hagedoorn P., Frijlink H.W. (2002). Characterization of inhalation aerosols: A critical evaluation of cascade impactor analysis and laser diffraction technique. Int. J. Pharm..

[8] Pilcer G., Amighi K. (2010). Formulation strategy and use of excipients in pulmonary drug delivery. Int. J. Pharm..

[9] Scheuch G., Siekmeier R. (2007). Novel approaches to enhance pulmonary delivery of proteins and peptides. J. Physiol. Pharmacol..

[10] Feeley J.C., York P., Sumby B.S., Dicks H. (1998). Determination of surface properties and flow characteristics of salbutamol sulphate, before and after micronisation. Int. J. Pharm..

[11] Telko M.J., Hickey A.J. (2005). Dry powder inhaler formulation. Respir. Care.

[12] Yamashita C., Ibaragi S., Fukunaga Y., Akagi A. (2008). Composition, Vessel, Dry Powder Inhalation System, and Related Methods for Transpulmonary Administration. U.S. Patent.

[13] Yamashita C., Fukunaga Y., Akagi A. (2010). Dry Powder Inhalation System for Transpulmonary Administration. U.S. Patent.

[14] Yamashita C., Matsushita H., Ibaragi S., Akagi A. (2010). Inhalation Device for Transpulmonary Administration. U.S. Patent.

[15] Weers J.G., Miller D.P. (2015). Formulation Design of Dry Powders for Inhalation. J. Pharm. Sci..

[16] Jehangir M., Ahmed M., Shafiq M.I., Samad A. (2017). Iftikhar-Ul-Haq UHPLC-PDA Assay for Simultaneous Determination of Vitamin D3 and Menaquinone-7 in Pharmaceutical Solid Dosage Formulation. J. Anal. Methods Chem..

[17] Mathew E.M., Moorkoth S., Rane P.D., Lewis L., Rao P. (2019). Cost-effective HPLC-UV method for quantification of Vitamin D2 and D3 in dried blood spot: A potential adjunct to newborn screening for prophylaxis of intractable paediatric seizures. Chem. Pharm. Bull..

[18] Oberson J.M., Bénet S., Redeuil K., Campos-Giménez E. (2020). Quantitative analysis of vitamin D and its main metabolites in human milk by supercritical fluid chromatography coupled to tandem mass spectrometry. Anal. Bioanal. Chem..

[19] Shekunov B.Y., Chattopadhyay P., Tong H.H.Y., Chow A.H.L. (2007). Particle Size Analysis in Pharmaceutics: Principles, Methods and Applications. Pharm. Res..

[20] Mitchell J., Nagel M. (2004). Particle Size Analysis of Aerosols from Medicinal Inhalers. KONA Powder Part. J..

[21] Miyamoto K., Taga H., Akita T., Yamashita C. (2020). Simple Method to Measure the Aerodynamic Size Distribution of Porous Particles Generated on Lyophilizate for Dry Powder Inhalation. Pharmaceutics.

[22] U.S. Department of Health and Human Services Food and Drug Administration Center for Drug Evaluation and Research Estimating the Maximum Safe Starting Dose in Initial Clinical Trials for Therapeutics in Adult Healthy Volunteers. https://www.fda.gov/regulatory-information/search-fda-guidance-documents/estimating-maximum-safe-starting-dose-initial-clinical-trials-therapeutics-adult-healthy-volunteers.

[23] Patel S.M., Nail S.L., Pikal M.J., Geidobler R., Winter G., Hawe A., Davagnino J., Rambhatla Gupta S. (2017). Lyophilized Drug Product Cake Appearance: What Is Acceptable?. J. Pharm. Sci..

[24] Edwards D.A., Ben-jebria A., Langer R. (1998). Recent advances in pulmonary drug delivery using large, porous inhaled particles. J. Appl. Physiol..

[25] Claus S., Schoenbrodt T., Weiler C., Friess W. (2011). Novel dry powder inhalation system based on dispersion of lyophilisates. Eur. J. Pharm. Sci..

[26] Claus S., Weiler C., Schiewe J., Friess W. (2013). Optimization of the fine particle fraction of a lyophilized lysozyme formulation for dry powder inhalation. Pharm. Res..

[27] Ohori R., Kiuchi S., Sugiyama S., Miyamoto K., Akita T., Yamashita C. (2020). Efficient optimization of high-dose formulation of novel lyophilizates for dry powder inhalation by the combination of response surface methodology and time-of-flight measurement. Int. J. Pharm..

[28] Kaialy W. (2016). A review of factors affecting electrostatic charging of pharmaceuticals and adhesive mixtures for inhalation. Int. J. Pharm..

[29] Mitaku S., Hirokawa T., Tsuji T. (2002). Amphiphilicity index of polar amino acids as an aid in the characterization of amino acid preference at membrane–water interfaces. Bioinformatics.

[30] Rau J.L. (2005). The inhalation of drugs: Advantages and problems. Respir. Care.

[31] Fukumoto K., Yoshizawa M., Ohno H. (2005). Room temperature ionic liquids from 20 natural amino acids. J. Am. Chem. Soc..

[32] Miyamoto K., Ishibashi Y., Akita T., Yamashita C. (2021). Systemic Delivery of hGhrelin Derivative by Lyophilizate for Dry Powder Inhalation System in Monkeys. Pharmaceutics.

[33] Griffith E.C., Vaida V. (2013). Ionization state of L-Phenylalanine at the Air—Water Interface. J. Am. Chem. Soc..

[34] Watry M.R., Richmond G.L. (2002). Orientation and Conformation of Amino Acids in Monolayers Adsorbed at an Oil/Water Interface As Determined by Vibrational Sum-Frequency Spectroscopy. J. Phys. Chem. B.

