# Supplementary material for: Optimization of Very Low-Dose Formulation of Vitamin D3 with Lyophilizate for Dry Powder Inhalation System by Simple Method Based on Time-of-Flight Theory
Source: Pharmaceutics. 2021 Apr 29;13(5):632. doi: 10.3390/pharmaceutics13050632 (PMC8145348; doi:10.3390/pharmaceutics13050632)
Supplement: Supplementary file 1 [file pharmaceutics-13-00632-s001.zip › pharmaceutics-1201921-supplementary.pdf]

pharmaceutics

Supplementary Materials: Optimization of Very Low-Dose Formulation of Vitamin D3 with Lyophilizate for Dry Powder Inhalation System by Simple Method Based on Time-of-Flight Theory

Kahori Miyamoto, Misato Yanagisawa, Hiroaki Taga, Hiromichi Yamaji, Tomomi Akita and Chikamasa Yamashita

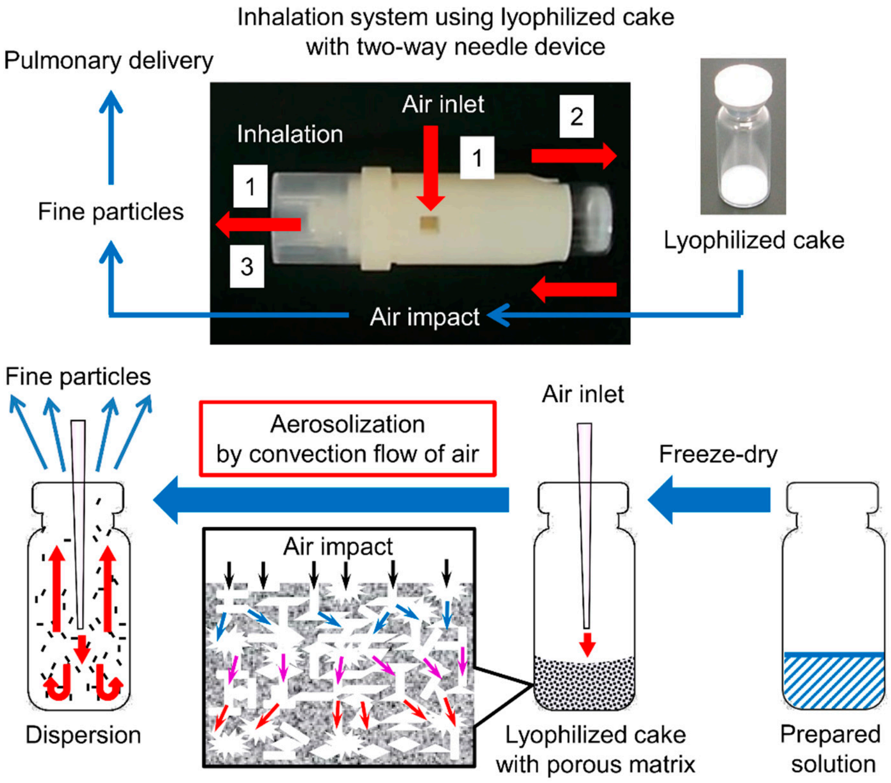

Figure S1. Lyophilizate for dry powder inhalation (LDPI) system. In the LDPI system, a freeze-dried cake is broken into particles suitable for pulmonary administration by air impact. (1) Air inlet through the two-way needle device occurs in synchronization with the patient's inhalation. (2) Aerosolization by the convection flow of air in the vial follows after disintegration of lyophilizate with a porous matrix structure by air impact. (3) Pieces of lyophilizate are reconstructed into particles suitable for pulmonary administration and then emitted from the vial through the device. Adapted from [1], MDPI, 2020.

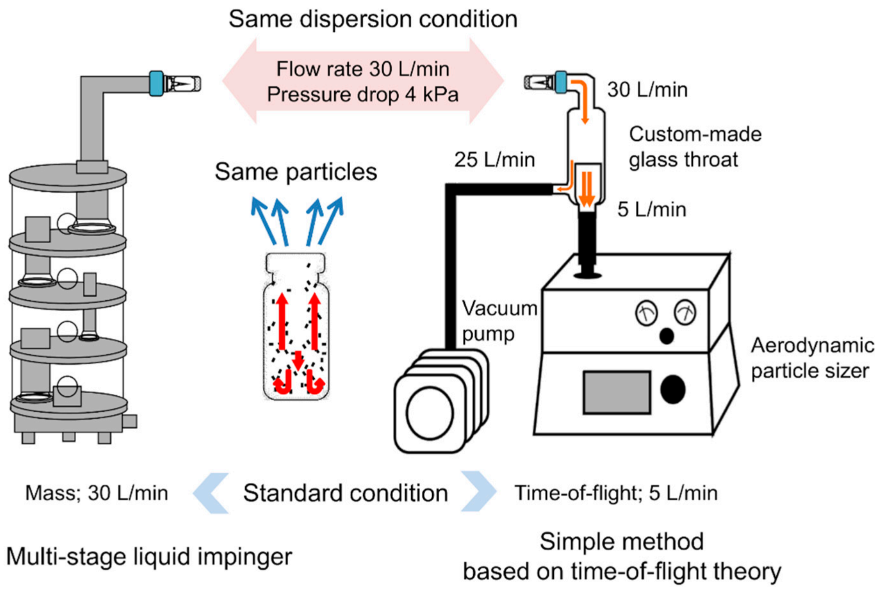

Figure S2. Measuring methods for the aerodynamic particle size distribution of LDPI formulations: multi-stage liquid impinger (MSLI) and our simple method based on time-of-flight (TOF) theory. Formulations are dispersed at flow rate of 30 L/min at a pressure drop of 4 kPa with the two-way needle device in both methods. This dispersion condition is the same as the standard condition for MSLI measurement. After dispersion, the aerodynamic size distribution of particles generated under the same condition is measured based on each theory. MSLI measurement is performed at a flow rate of 30 L/min and the aerodynamic particle size distribution was calculated by quantification of main drugs. In the simple method based on TOF theory, a custom-made glass throat and a vacuum pump are added to set the same dispersion condition as that for MSLI measurement, and the aerodynamic particle size distribution is measured using an aerodynamic particle sizer at a flow rate of 5 L/min by TOF.

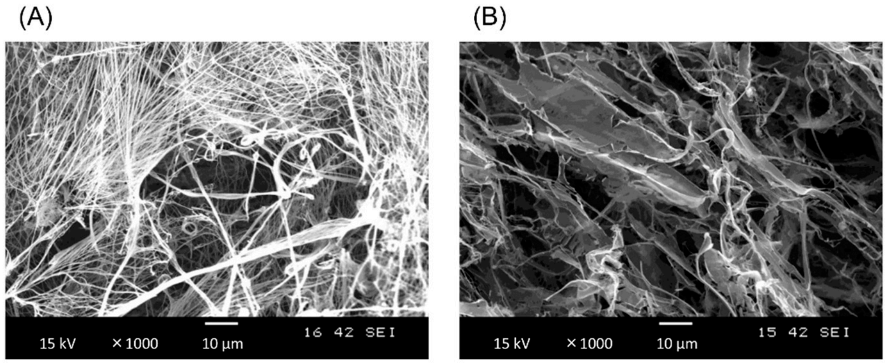

Figure S3. Microstructure of the placebo formulation containing 0.5 mg/vial of phenylalanine or methionine observed by scanning electron microscope (SEM). SEM images of phenylalanine (**A**) and methionine (**B**) are shown. Both formulations showed a porous matrix but microstructure was different. Phenylalanine which showed high FPF had a fiber-like network structure whereas methionine which showed low FPF had a thin layer structure.

References

1. Miyamoto, K.; Taga, H.; Akita, T.; Yamashita, C. Simple Method to Measure the Aerodynamic Size Distribution of Porous Particles Generated on Lyophilizate for Dry Powder Inhalation. *Pharmaceutics* **2020**, *12*, 976, doi:10.3390/pharmaceutics12100976.
